# Multimodal management of solitary axillary lymph node metastasis from a recurrent nasopharyngeal undifferentiated carcinoma: a case report and review of the literature

**DOI:** 10.3389/fonc.2025.1578547

**Published:** 2025-06-05

**Authors:** Houyem Mansouri, Ines Zemni, Samia Ben Hssine, Sabrine Boukhris, Leila Achouri, Najet Mahjoub

**Affiliations:** ^1^ Department of Surgical Oncology, Regional Hospital of Jendouba, Faculty of Medicine of Tunis, University of Tunis El Manar, Jedouba, Tunisia; ^2^ Department of Surgical Oncology, Salah Azaiez Institute of Oncology, Faculty of Medicine of Tunis, University of Tunis El Manar, Tunis, Tunisia; ^3^ Department of Radiation Therapy, Regional Hospital of Jendouba, Faculty of Medicine of Tunis, University of Tunis El Manar, Jedouba, Tunisia; ^4^ Department of Medical Oncology, Regional Hospital of Jendouba, Faculty of Medicine of Tunis, University of Tunis El Manar, Jedouba, Tunisia

**Keywords:** recurrence, radiotherapy, chemotherapy, nasopharyngeal carcinoma, axillary lymph node metastasis

## Abstract

**Introduction:**

Undifferentiated Nasopharyngeal carcinoma (UNPC) exhibits the highest loco-regional cervical lymph node recurrence among head and neck epithelial malignancies. Axillary lymph node recurrence in NPC is uncommon.

**Case description:**

A 44-year-old male presented with two-month history of painless swelling of the right axilla. He was diagnosed with stage IVB UNPC three years and eight months previously and had undergone neoadjuvant chemotherapy, and concurrent chemo-radiotherapy. Biopsy of the axillary swelling revealed metastatic UNPC. The patient was treated with axillary lymph node dissection followed by chemotherapy, and right axillary radiotherapy. Currently, the patient has been disease free for 12 months.

**Conclusion:**

To our knowledge, this is the second reported case of UNPC with isolated axillary lymph node recurrence after remission, treated with axillary lymph node dissection, chemotherapy, and radiotherapy. This case highlights a rare pattern of metastasis and underscores the need for thorough investigations and imaging in the long-term follow-up of NPC.

## Introduction

Nasopharyngeal carcinoma (NPC) is a malignant epithelial tumor of the nasopharynx characterized by a markedly high incidence in certain geographic and ethnic groups. The highest rates are found among populations from Southern China, while regions such as Southeast Asia, North Africa, and the Inuit communities in Canada and Alaska show intermediate levels of incidence (1, 2). NPC is typically diagnosed at an advanced local stage, with lymph node metastases in 90% of patients. Approximately 5–10% of patients develop metastases, and when the cancer spreads to critical organs and tissues, the prognosis becomes extremely poor (3). Although chemoradiotherapy (CRT) provides effective locoregional control, 15–18.5% of newly diagnosed patients without initial distant metastases ultimately experience therapeutic failure due to tumor cell metastasis after treatment (4). Common sites of distant metastases are bone, liver, and lung, while metastases to axillary lymph nodes are extremely rare. In most cases, these are either extensive distant metastases or a recurrence at the site of the primary tumor (5). Advances in IMRT (Intensity-Modulated Radiation Therapy) and imaging technologies have significantly improved local control of NPC by enhancing tumor delineation and allowing more precise identification of organs at risk. However, the management of distant metastases remains a major challenge for NPC patients, with no established consensus on optimal treatment strategies (6). We describe a case of solitary axillary lymph node recurrence in a patient with NPC, treated by axillary lymph node dissection followed by chemotherapy and radiation therapy. This report aims to draw attention to a rare but possible site of NPC recurrence, emphasizing the importance of early diagnosis and the initiation of comprehensive treatment strategies.

## Case description

A 44-year-old male presented in October 2023 with a two-month history of painless right axillary swelling. He had previously been diagnosis with stage IVB (T1N3M0) undifferentiated nasopharyngeal carcinoma (UNPC), initially identified in January 2020, with right cervical lymphadenopathy as the only symptom.

At that time, nasal endoscopic examination revealed a mass in the right Rosenmüller fossa and biopsy confirmed the diagnosis of undifferentiated non-keratinizing squamous cell carcinoma of the nasopharynx. Pre-treatment magnetic resonance imaging (MRI) revealed an expansive lesion involving the posterior, medial and right paramedian walls of the nasopharynx, with filling of the right Rosenmüller fossa. The lesion spared the retro- and parapharyngeal spaces and the choanae, with no evidence of extension to the skull base. Associated adenopathies included right paramedian retropharyngeal nodes, bilateral level II cervical lymph nodes, and right level IV lymph nodes. The contrast Enhanced Computed Tomography (CECT) scan of the brain, thorax and abdomen showed no distant metastasis. He received four cycles of neoadjuvant chemotherapy with gemcitabine (1,250 mg/m²) and cisplatin (80 mg/m²), followed by concurrent chemoradiotherapy (CCRT). Radiotherapy was delivered by the volumetric modulated arc therapy (VMAT) technique using 6 MV photons, administering a total dose of 70 Gy in 33 fractions over a seven week period. Concurrent chemotherapy consisted of weekly cisplatin (40 mg/m²).

**Figure 1:**
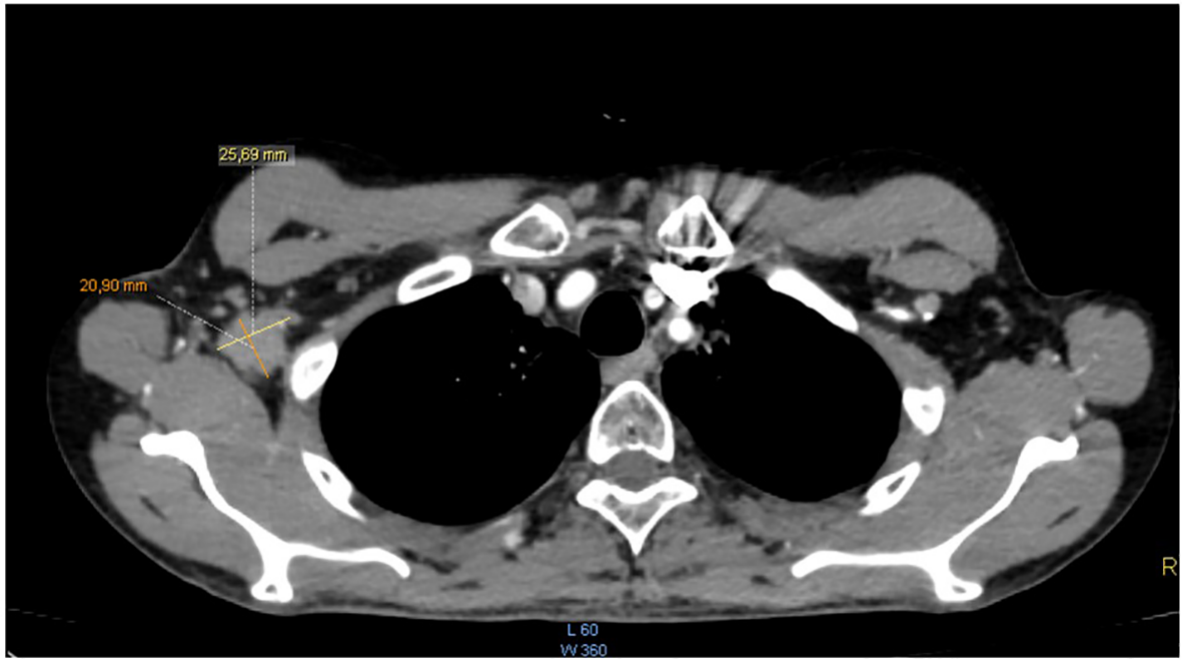
Axial CT scan with contrast injection: right axillary adenopathy measuring 25*20mm.

**Figure 2:**
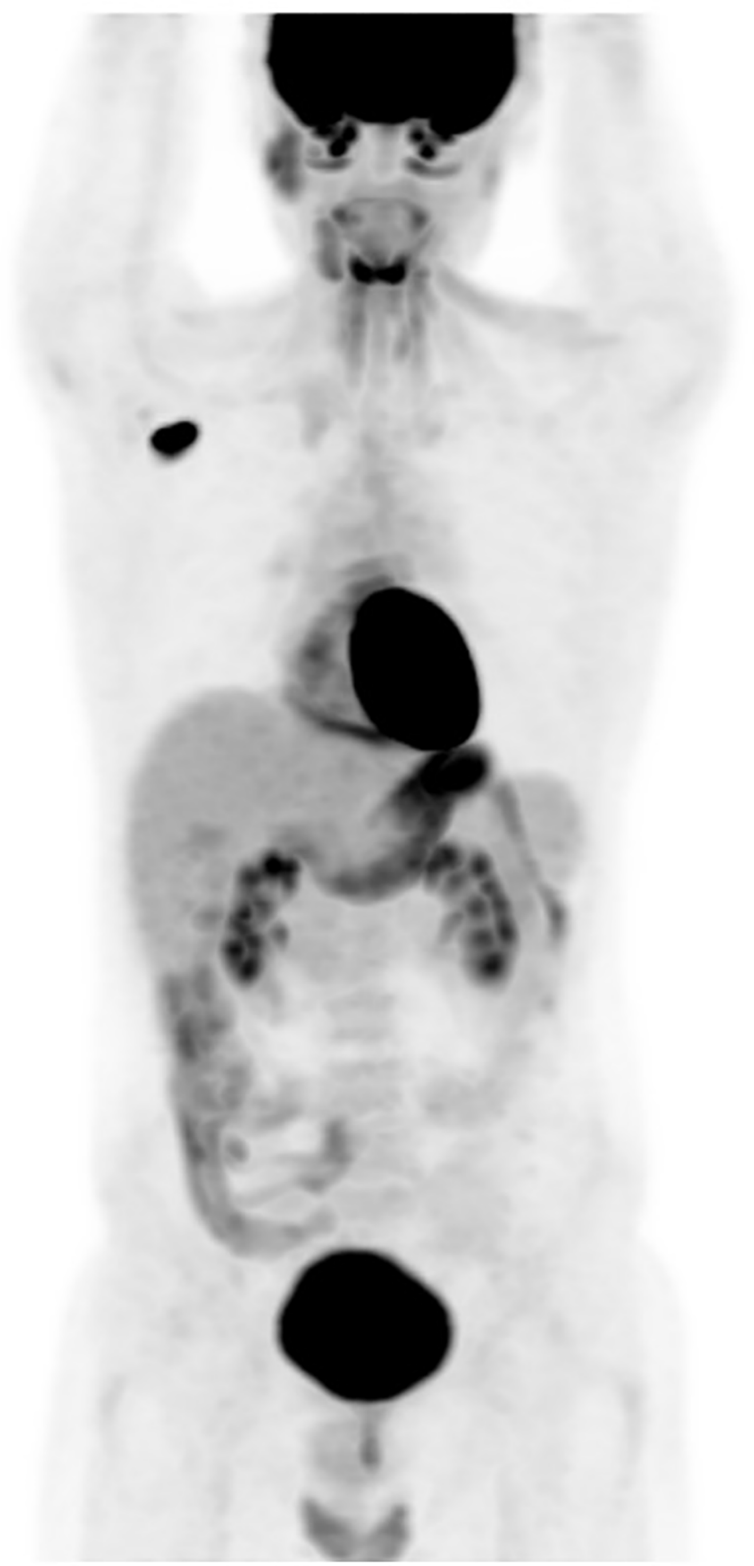
(PET-CT) scan: an isolated hypermetabolic focus in right enlarged axillary lymph node with SUV max of 13.4.

The patient completed the therapeutic protocol in February 2021. Post-treatment assessments—including clinical and endoscopic examinations and an MRI performed three months later—confirmed complete remission, with no evidence of residual disease in the nasopharynx or cervical lymphatic regions. Two years and eight months after completing treatment, the patient presented with increasing, painless swelling of the right axilla without associated auditory or nasal symptoms. Physical examination revealed a firm, immobile axillary mass measuring 35 × 20 mm, with no palpable cervical lymphadenopathy. Systemic examination was otherwise unremarkable, and nasal endoscopy demonstrated no masses in the nasopharynx. Axillary ultrasound demonstrated a hypoechoic, oval right axillary lymph node with lobulated contours and a preserved fatty hilum, measuring 35 × 19 mm, accompanied by hyperechoic infiltration of the surrounding fat. Additionally, a second subpectoral lymph node with similar characteristics measured 25 × 16 mm. The left axillary region and supraclavicular fossae were unremarkable. A surgical excisional biopsy of the right axillary lymphadenopathy was undertaken to obtain a sufficient tissue sample for comprehensive histopathological analysis, including detailed assessment of lymph node architecture, in order to exclude alternative etiologies such as lympho-proliferative malignancies. The histopathological examination revealed features similar to those of the initial nasopharyngeal biopsy, suggesting metastasis from a keratinizing nasopharyngeal carcinoma. The contrast-enhanced computed tomography (CECT) of the brain, neck, thorax, and abdomen revealed no enhancing mass in the nasopharynx or enlarged cervical lymph nodes, but demonstrated an enlarged right axillary lymph node (**Figure 1**), with no evidence of distant metastases. MRI likewise showed no signs of local nasopharyngeal recurrence. The 18F-FDG PET/CT revealed an isolated hypermetabolic focus in the right axillary lymph node (SUVmax 13.4) without abnormal uptake elsewhere in the chest, abdomen, or pelvis (**Figure 2**).

**Figure 3:**
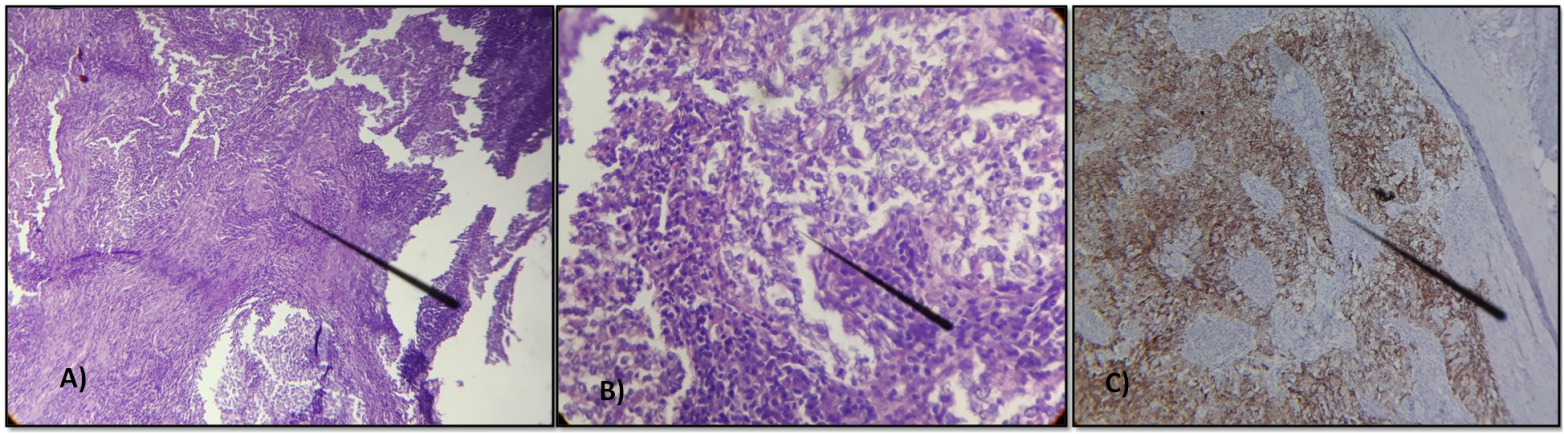
Lymph node metastasis of an undifferentiated carcinoma arranged in lobules, cords, or sometimes syncytial masses. The tumor cells have indistinct borders with vesicular nuclei and prominent nucleoli, occasionally mitotic. **(A)** Hematoxylin x200 **(B)** Hematoxylin x400 **(C)** Immunohistochemical study showing the positivity of the tumor cells for cytokeratin AE1/AE3.

The patient underwent a right axillary dissection, and histological examination revealed the presence of 12 whitish metastatic lymph nodes, measuring between 0.3 and 3 cm, infiltrated by a carcinomatous proliferation exhibiting trabecular, lobular, or syncytial architecture. The cells were large, with indistinct borders and slightly eosinophilic cytoplasm, and exhibited markedly atypical vesicular nuclei with prominent nucleoli and occasional mitotic figures (**Figures 3A, B**). An immunohistochemical study showed the positivity of the tumor cells for cytokeratin AE1/AE3 (**Figure 3C**). Postoperatively, the patient received 3 cycles of chemotherapy according to the TPF protocol, consisting of 5-FU (750 mg/m² by 24-hour continuous infusion for 5 days), Cisplatin (75 mg/m² on Day 1), and Docetaxel (75 mg/m² on Day 1) every 3 weeks, followed by right axillary radiotherapy at a dose of 50 gray over 25 fractions. The patient completed the prescribed multimodal treatment protocol (surgery, chemotherapy, and radiotherapy) without interruption, and no significant adverse or unanticipated events were reported during or after treatment. Currently, the patient has remained disease-free for 12 months, as confirmed by regular clinical and endoscopic examinations, together with contrast-enhanced computed tomography (CECT) of the brain, neck, thorax, and abdomen, and MRI—imaging studies that were initiated three months after treatment completion.

## Discussion

NPC is a malignant epithelial tumor of the nasopharynx, with a notably higher incidence in certain geographic and ethnic groups. It is often diagnosed at an advanced stage, with lymph node metastases present in 90% of cases and distant metastases in 5–10% of patients (3). Despite the effective locoregional control provided by chemoradiotherapy, 15–18.5% of newly diagnosed patients without initial distant metastases end up suffering treatment failure due to tumor cell metastases following treatment (4). A recent retrospective study of 2599 NPC patients revealed that 59% of recurrent NPC cases occurred within the first two years after treatment ended (7). The most frequent recurrence were local (73.5%), both local and regional cervical lymph nodes (21.7%), and distant metastasis (6.6%) (8). Common sites of distant metastases include bone, liver, and lungs, while axillary lymph nodes metastases are extremely rare. Most reported cases were associated with widespread distant metastases or recurrence at the primary tumor site (5). In fact, Memorial Sloan-Kettering Cancer Center reported 85 cases of axillary lymph node metastases from non-mammary primary sites between 1990 and 2010. Of these cases, 8% were from head and neck cancers, although no cases of NPC were reported (9).

We present a rare case of an isolated axillary recurrence of a NPC within the second two years of follow up. We conducted a literature review to identify similar cases of axillary lymph node metastases in patients with nasopharyngeal carcinoma (NPC). A comprehensive search was performed using the PubMed, Scopus, science direct and Google Scholar databases for articles published up to March 2024 using the following keywords: “nasopharyngeal carcinoma,” “axillary lymph node,” “metastasis,” “18FDG PET/CT; radiotherapy”, “chemotherapy” and “recurrence”.” Boolean operators such as “AND” and “OR” were used to refine the search. We included case reports and case series describing axillary lymph node metastasis in patients with histologically confirmed NPC, regardless of the language of publication. Articles written in English, and French were considered, provided they contained sufficient clinical detail. The reference lists of included articles were also manually reviewed to identify additional relevant studies. All selected cases are summarized in **Table 1**.

**Table 1:** Cases of axillary metastasis from NPC described in the literature.

Publications/ years	N° cases	Age/ sex	Primary tumor characteristic	Previous treatment	Meta /synch	Time to axillary metastasis (months)	Isolated axillary metastasis	Treatment of axillary metastasis	Outcomes survival
Thrirumalarairaj Et al (32) 1991	1 case	36, M	SCC Unknown stage	RT	Meta	96	Yes	RT	Complete remission
Koch et al. (10) 1998	1 case	47, M	SCC Unknown stage	Right neck LND + RT	Meta	7	Yes	Axillary LND +RT	–
Lee et al. (18) 2005	1 case	52, M	UNPC T1N2M0	CRTCT	Meta	5	No: Local recurrence Inferior spinal LN	Axillary LND	-
Yazici et al. (15) 2013	1 case	48, M	UNPC	CT	Syn	–	No: bilateral cervical LN Bone metastasis	CT	Axillary and Hepatic progression during 4 months with complete remission for 2 years after CT
Yong Tae Hong et al. (11) 2016	1 case	45, M	UNPC T2N3bM0 (stage IVB)	NACT+ CRTCT + Neck LND + ADJ CT	Meta	3	No: Supra-clavicular area + posterior neck LN	CT	Alive under treatment
Kang et al. (19) 2016	1 case	64, W	SCC T4N1M0	RTCT + LND for cervical LN recurrence	Meta	132	Yes	Not described	–
Kuo et al. (5) 2017	1 case	66, M	UNPC T2N2M0	NACT+CRTCT	Meta	84	Yes	CT (Epirubicin, cisplatin, Leucovorin, and 5-FU) + RT	Remission 17 months
Subha et al. (20) 2017	1 case	44, M	UNPC T2N3bM0	CRTCT	Meta	36	No: Local recurrence Mediastinal LN Supra-clavicular LN	Palliative CT	–
Mohammad Nasir et Subha (8) 2019	1 case	44,M	Non- keratinizing nasopharyngeal carcinoma T2N3M0	NACT+CRTCT	Meta	24	No: anterior mediastinal LN	Palliative CT: Carboplatin + Gemcitabine (4 cycles)	Died: progression
Oprean et al. (30) 2021	1 case	40,W	Non−keratinizing nasopharyngeal SCC T1N2M0	NACT+CRTCT	Meta	24	No: supra-clavicular LN	NACT: Carboplatine + Cisplatine + Surgical LN Excision + adjuvant RT	Complete remission
Hajra et al. (33) 2023	1 case	22,M	UNPC Unknown stage	-	Syn	-	No: Mediastinal, abdominal, and cervical LN. Pleural, hepatic, splenic, and bone metastasis.	-	-
Li et al. (12) 2023	6 cases	68,M 29,W 41,M 61,M 36, M 59, M	UNPC, T3N3M0 UNPC, T2N3M0 UNPC, T3N3M0 UNPC, T1N3M0 UNPC T4N3M0 UNPC, T3N3M0	NACT+CRTCT NACT+CRTCT NACT+CRTCT NACT+CRTCT + ADJ CT NACT+CRTCT + ADJ CT NACT+CRTCT + ADJ CT	Meta Meta Meta Meta Meta Meta	22.5 53.5 4 17.7 30 62.4	Yes Yes Yes Yes Yes Yes	Palliative CT Palliative CT Palliative CT Palliative CT Palliative CT Palliative CT	- - - - - -
Guan et Yunus (22) 2024	3 cases	39; M 74; F 58; F	T1N2M0 Unknown stage T2N1M0	RT RT RT	Meta Meta Meta	31 36 67	No: neck recurrence No: LR recurrence No: lung+ bone metastasis	Palliative CT RT palliative CT Palliative CT	Alive, 1 year Progressive metastasis Died at 18 months
Musa et al. (34) 2024	1 case	55,M	T2N3M0	CRTCT	Meta	9	No: LR recurrence Mediastinal and cervical LN. pulmonar, hepatic, and splenic metastasis	Palliative CT	Died: progression

M, male; F, female; Meta, Metachronous, Syn, Synchronous; SCC, squamous cell carcinoma; NACT, neoadjuvant chemotherapy; CRTCT, Concurrent chemo-radiation therapy; RT, radiation therapy; CT, chemotherapy; ADJ CT, adjuvant chemotherapy; LR, locoregional; LN, lymph node; LND, lymph node dissection.

Typically, the lymphatic drainage of the nasopharynx primarily flows to the cervical lymph nodes, while the lymphatic system in the axilla drains from the chest wall and the distal parts of the upper limb toward the subclavian venous system, following the path of the axillary vein (10). The precise mechanism of axillary lymph node metastases from head and neck carcinoma remains unclear, though several hypotheses have been proposed. Firstly, cancer cells could have spread distally through the lymphatic system, from the thoracic duct to the axillary lymph nodes (11). Secondly, the fat in level V is continuous with axillary fat, and cancer cells might have spread via this pathway. However, our patient did not present with positive nodes at level V, either initially or at the time of recurrence, which does not support this possible route of spreading. Yet, the study by Li et al. suggested that patients with supraclavicular lymph node metastasis prior to treatment, regardless of group V, are more likely to develop axillary lymph node metastasis afterward, with the metastasis primarily occurring on the same side as the clavicular lymph node involvement or the more severely affected side (12). This was the case for our patient, who had initial right cervical lymph involvement and presented with right axillary recurrence. Nevertheless, our patient had been treated with concurrent CRT, and several studies have suggested that patients with significant prior alterations to their lymphatic drainage pathways—whether through lymphadenectomy, tumor recurrence, or prior radiation therapy—may experience reduced blood supply in the area surrounding the nasopharynx, creating conditions less favorable for tumor growth. As a result, the tumor may have migrated to a distant location, such as the axilla (13, 14).

It is widely recognized that more common causes, such as lymphoma, must be excluded when evaluating patients with axillary lymph node involvement. However, it is equally important to consider accompanying signs and symptoms for a precise diagnosis (15). In our case, the absence of auditory or nasal symptoms, along with the lack of cervical lymphadenopathy in typical NPC recurrence sites—and the absence of locoregional recurrence confirmed by regular radiological imaging and endoscopy—warranted an axillary lymph node biopsy. This was necessary to rule out lymphoma by providing an adequate tissue sample for comprehensive histopathological analysis and allowing proper evaluation of lymph node architecture, which is a key factor in the accurate identification and classification of lymphoid malignancies—something that cannot be achieved through fine-needle aspiration cytology (FNAC). Moreover, it has been demonstrated that advanced TNM stages, particularly stage IV and N3, are at high risk of recurrence and particularly axillary lymph node metastasis (8, 12).

Recently, positron emission tomography (PET) imaging and the quantification of plasma Epstein–Barr virus (EBV) DNA have demonstrated promising effectiveness in the staging, treatment response assessment, and detection of relapse in NPC (5). In fact, positron emission tomography with 18F-fluorodeoxyglucose (FDG-PET) is a valuable tool for identifying local recurrences and distant metastases, offering superior detection capabilities compared to traditional anatomical imaging, with a combined sensitivity of 95% and specificity of 90% (16, 17). PET/CT may play a significant role in managing NPC by providing whole-body imaging to identify additional metastatic sites. The contribution of FDG-PET in assessing disease extent during axillary nodal recurrence of NPC has been reported by six teams (5, 8, 11, 18–20). In two cases, it confirmed the isolated nature of the recurrence (5, 19), a finding that was also observed in our patient.

The recent meta-analysis of Alami et al. suggested that high levels of cell-free EBV DNA prior to chemo-radiotherapy are associated with poor prognosis. Furthermore, the presence of detectable cell-free EBV DNA at the end of treatment is a strong predictor of disease recurrence and distant metastasis (21). Unfortunately, Epstein–Barr virus testing was not implemented as a complementary surveillance tool alongside conventional imaging for monitoring treatment failure at our institution.

In current clinical practice, axillary lymph node involvement in nasopharyngeal carcinoma (NPC) is classified as distant metastasis, typically considered incurable and associated with a poor prognosis (22). Moreover, distant metastases remain a major challenge for NPC patients due to the lack of an established consensus on the optimal treatment strategies. Given the rarity of axillary metastases as a form of distant recurrence from head and neck tumors, it is evident that there is no definitive agreement on the best approach to their management.

According to the NCCN (National Comprehensive Cancer Network) guidelines, systemic chemotherapy remains the primary treatment approach for managing patients with metastatic NPC. As reported in previous studies, metastatic NPC is most commonly treated with platinum-based chemotherapy, achieving an overall response rate ranging from 50% to 90% (5). Currently, gemcitabine combined with cisplatin is recognized as the standard first-line chemotherapy for recurrent or metastatic NPC, based on the results of a randomized Phase III clinical trial (23). In addition, taxane combined with cisplatin—such as the TPF or TP regimen—has also been proven effective in advanced, recurrent, and metastatic NPC (24, 25). We administered a Taxane/Cisplatin-based chemotherapy regimen to our patient, who had previously received Gemcitabine combined with Cisplatin as neoadjuvant therapy during the treatment of the primary tumor.

Nevertheless, the concept of oligometastasis, introduced in 1995, described an intermediate stage where cancer has spread to a limited number of distant sites (26, 27). This concept suggests that a subset of patients with oligometastasis from NPC may achieve long-term survival following aggressive local treatments, including chemotherapy, surgery, or definitive radiotherapy to the metastatic sites (28, 29). It is recognized that patients with metastatic NPC do not represent a homogeneous group, and individual differences exist both within the group and between patients in terms of treatment response. It is therefore reasonable to suggest that an individualized predictive model is necessary to identify the factors associated with survival, which could in turn support the development of more personalized treatment strategies. In this context, Peng et al. developed a prognostic nomogram to identify NPC patients with metachronous metastases who are most likely to benefit from local treatments (6). The proposed model incorporates several prognostic variables, including sex (female vs. male), disease-free interval (>12 months vs. ≤12 months), presence of liver metastases, number of metastatic lesions (1 vs. 2–5 vs. >5), presence of locoregional recurrence, and number of palliative chemotherapy cycles administered (≥4 vs. <4). Based on the median total score derived from the nomogram, patients were stratified into low-risk (score 0–122) and high-risk (score 123–260) groups. Notably, the study demonstrated that patients classified as low-risk who received palliative chemotherapy in combination with local treatment exhibited significantly improved survival outcomes compared to those treated with palliative chemotherapy alone (HR = 0.570; 95% CI = 0.343–0.947; p=0.030) (6). According to this nomogram, our patient was classified in the low-risk group, with a total score of 24 attributed to male sex as the only risk factor. This stratification supported the rationale for selecting an aggressive multimodal therapeutic approach in his case.

Currently, there is insufficient evidence to assess the efficacy of axillary lymph node dissection as a surgical treatment for isolated axillary lymph node metastases (8) and the majority of reported cases of axillary metastasis from NPC in the literature have been managed with palliative chemotherapy alone (**Table 1**). Some authors have suggested that axillary dissection may be used as a salvage treatment, with or without adjuvant radiotherapy, in cases where further metastatic evaluation confirms the absence of other sites of tumor recurrence (22). Koch et al. reported the oncological outcomes of four cases of axillary recurrences of head and neck cancers: one case of squamous cell carcinoma (SCC) of the left arytenoid and aryepiglottic fold, one case of SCC of the epiglottis, one case of SCC of the supraglottic larynx, and one case of SCC of the nasopharynx. All four patients were treated with axillary lymph node dissection, followed by adjuvant axillary radiotherapy in the last case (10). Lee et al. described undifferentiated nasopharyngeal carcinoma with axillary lymph node involvement as a component of failure following chemoradiotherapy, treated with axillary lymph node dissection without additional treatment (18). Operan et al. described a case of axillary recurrence of an initially unresectable nasopharyngeal carcinoma, treated with chemotherapy followed by surgical excision of the axillary recurrence and adjuvant axillary radiotherapy. This approach led to complete remission and highlighted the potential role of axillary surgery in improving local control when (30). In our case as well, the multimodal approach combining chemotherapy for systemic control, along with surgery and radiotherapy, achieved complete remission. Regardless of the location of the oligometastasis, the role of surgery within the therapeutic approach remains a subject of debate.

Moreover, in our case, while pre-op CECT and PET scan revealed an isolated lymph node, post-operative histopathology showed 12 metastatic lymph nodes. The discrepancy between pre-operative imaging findings and post-operative histopathology can be attributed to several factors. While the CECT and PET scans identified an isolated lymph node, it is important to consider the limitations of imaging techniques in detecting microscopic or small-volume metastases, especially in the context of axillary lymph node involvement. Histopathological examination, which provides a more detailed and comprehensive assessment of the lymph nodes, revealed additional metastatic nodes that were not detected on imaging. This highlights the importance of combining imaging and histopathological analysis in the accurate staging and assessment of metastatic disease. The identification of these additional metastatic nodes underscores the need for thorough surgical resection and histopathological evaluation, even when imaging suggests a more localized disease.

The comparison between surgery combined with systemic therapy and systemic therapy alone, or between surgery combined with systemic therapy and surgery alone, for oligometastatic NPC remains largely unexplored. The primary objective is to determine the most effective approach to optimize outcomes for patients with oligometastatic NPC (31).

This case report provides a comprehensive and well-documented description of an extremely rare presentation of nasopharyngeal carcinoma (NPC) with isolated axillary lymph node recurrence. It includes a detailed clinical narrative, supported by imaging findings (CT, PET-CT, and MRI), histopathological evidence, and treatment outcomes. Although our case is noteworthy, it is limited by the absence of Epstein–Barr virus (EBV) DNA quantification, which is a recognized prognostic and monitoring biomarker in NPC. Additionally, immunohistochemical profiling of the recurrence was not detailed extensively, which could have provided further diagnostic precision.

## Conclusion

To our knowledge, this is the second documented case in the literature of UNPC presenting with axillary lymph node involvement as an isolated manifestation of late recurrence following complete remission and treated with axillary lymph node dissection followed by chemotherapy and radiation therapy. This case highlights an uncommon pattern of lymphatic metastasis, with no evidence of supraclavicular, mediastinal of other distant disease on PET imaging. It underscores the importance of thorough physical examination and correlation with imaging modalities in the long-term monitoring of these patients, as it represents a rare presentation of recurrent nasopharyngeal carcinoma.

## Data Availability

The original contributions presented in the study are included in the article/supplementary material. Further inquiries can be directed to the corresponding author.
